# Case Report: Antifreeze Ingestion and Urine Fluorescence

**DOI:** 10.21980/J8G05T

**Published:** 2020-01-15

**Authors:** Taras Varshavsky, Meigra Myers Chin

**Affiliations:** * Rutgers Robert Wood Johnson Medical School, Department of Emergency Medicine, New Brunswick, NJ

## Abstract

This case report presents a patient with ethylene glycol intoxication from antifreeze ingestion. Ethylene glycol is an active ingredient in antifreeze, traditionally causing an anion gap metabolic acidosis with a high osmolality gap. In our emergency department, serum ethylene glycol is a send-out test requiring hours for a result. The patient presented here had an initial acidotic venous blood gas with an elevated serum osmolality and osmolality gap, and a normal anion gap. Quick bedside analysis of the patient’s urine using ultraviolet fluorescence gave supportive evidence for the ingested substance while the serum ethylene glycol level was still pending. The patient was promptly treated with fomepizole, pyridoxine, thiamine, and sodium bicarbonate. Several hours after admission, the ethylene glycol level had resulted as 636 mg/dL. Due to the quick initiation of treatment, the patient had no complications or signs of end-organ damage during admission.

**Topics:**

Ethylene glycol, fomepizole, toxicology, ultraviolet fluorescence.

[Fig f1-jetem-5-1-v29] [Fig f2-jetem-5-1-v29]

**Figure f1-jetem-5-1-v29:**
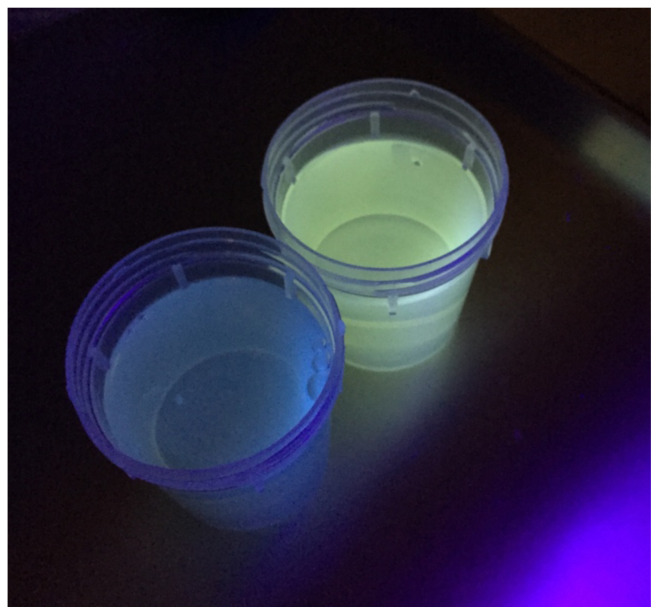


**Figure f2-jetem-5-1-v29:**
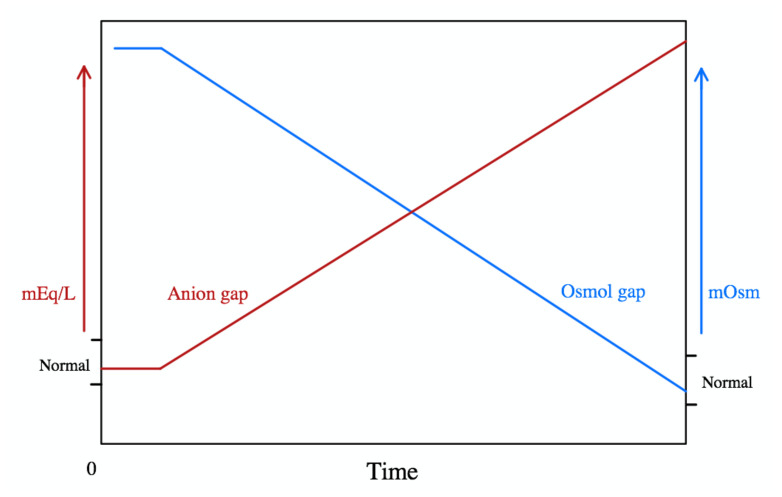


## Introduction

Ethylene glycol is a component of antifreeze, solvents, cleaners, and many other industrial products.^1^,^2^ It is metabolized by alcohol dehydrogenase (ADH) into toxic metabolites including glycolic acid, glyoxylic acid, and oxalic acid which are responsible for the organ damage associated with antifreeze ingestion. Symptoms can include nausea, vomiting, ataxia, abdominal pain, pulmonary edema, renal failure, stupor, and coma. As the toxic metabolites accumulate with time, the bicarbonate concentration will decrease resulting in an elevated anion gap and decreased osmolality gap.^3^ A severe anion gap metabolic acidosis occurs with ethylene glycol intoxication; therefore, early treatment is required to prevent permanent tissue damage.

## Presenting concerns and clinical findings

A 70-year-old male with a history of alcohol use disorder presented to the emergency department after a suicide attempt. The patient’s wife found an open bottle of whiskey and a gallon of antifreeze with a third of the fluid missing, which the patient admitted to drinking. On exam, the patient was alert and appeared intoxicated with a blood pressure of 122/62 mmHg, heart rate of 83 beats/min, respiratory rate of 16 breaths/min, oxygen saturation of 97% on room air, and temperature of 97.7^o^F. The patient had slurred speech and non-bloody emesis on his clothing, but otherwise the physical exam was unremarkable.

## Significant findings

The patient’s urine sample (right) was compared to a control (left) using a Wood’s lamp. It revealed light green fluorescence under ultraviolet light, which increased suspicion for ethylene glycol poisoning from antifreeze ingestion.

## Patient Course

The patient had stable vital signs while in the emergency department and maintained his altered mental status. His initial venous blood gas had a pH of 7.28, a pCO2 of 49 mmHg, and a serum bicarbonate of 24 mmol/L. His anion gap was within normal range at 15 mEq/L, but he had an elevated serum osmolality of 442 mOsm/kg, an elevated osmolality gap of 121.1 mOsm/kg, and an elevated lactate of 2.7 mmol/L. His glucose was 137 mg/dL, his Cr was 0.9 mg/dL, and his urine was negative for ketones. The patient’s ethanol level was 76 mg/dL, and the ethylene glycol level was later determined to be 636 mg/dL. He was initially treated in the emergency department with fomepizole, pyridoxine, thiamine, normal saline bolus, sodium bicarb 8.4% 100 mEq injection, and D5 with sodium bicarb 8.4% 100 mEq maintenance fluids. During his medical intensive care unit (MICU) hospitalization, the patient had no complications or signs of end-organ damage. His treatment included maintenance doses of fomepizole, pyridoxine, and thiamine. His osmolar gap trended downward and his ethylene glycol level decreased to 43 mg/dL at which point fomepizole, pyridoxine, and thiamine were stopped, and only supportive care was continued. The patient was hospitalized for a total of seven days and was voluntarily admitted to an inpatient psychiatric facility for further management.

## Discussion

The mainstay of treatment for ethylene glycol intoxication is early use of fomepizole, a competitive inhibitor of ADH, to prevent the formation of the toxic metabolites.^4^ Its early use is emphasized since ADH inhibition does not prevent toxicity if the metabolites are already formed. Ethylene glycol toxicity is delayed with co-ingestion of ethanol because ethanol is also a competitive inhibitor of ADH, though it is inferior to fomepizole.^5^ As shown in the graph, the patient most likely presented early after his toxic ingestion, correlating his high osmolality gap with his normal anion gap. However, by concurrently drinking whiskey with the antifreeze, the ethanol likely slowed the progression of an anion gap metabolic acidosis. Treatment with sodium bicarbonate helps with excretion of the acids in urine and decreases the penetration of the toxic metabolites into end-organ tissue.^2^ Quick initiation of treatment in this patient prevented a high anion gap metabolic acidosis during his hospitalization.

When history and clinical suspicion is high for ethylene glycol intoxication, treatment should not be delayed for a serum ethylene glycol level. At this emergency department, it is a send-out lab that takes hours for a result to return, by which time the patient had already been admitted to the MICU. The quick bedside analysis of the patient’s urine in the emergency department using a Wood’s lamp showed light green fluorescence which indicated the likely presence of fluorescein, supporting the history of antifreeze ingestion and subsequent ethylene glycol intoxication. Fluorescein is added to most antifreeze solutions to detect radiator leaks under UV light.^6^ However, the presence or absence of urine fluorescence needs to be interpreted with caution. Fluorescence may appear only transiently after ingestion and cause false negatives.^7^,^8^ False positives can also occur, since fluorescence can appear in normal urine or may be secondary to certain food or toxic substances unrelated to ethylene glycol. However, in the setting of the proper clinical context, this was a helpful aid in the bedside confirmation of ethylene glycol intoxication.

## Supplementary Information




