# Supplementary figures and images for: Case Report: Antifreeze Ingestion and Urine Fluorescence
Source: J Educ Teach Emerg Med. 2020 Jan 15;5(1):V29–31. doi: 10.21980/J8G05T (PMC10332530; doi:10.21980/J8G05T)

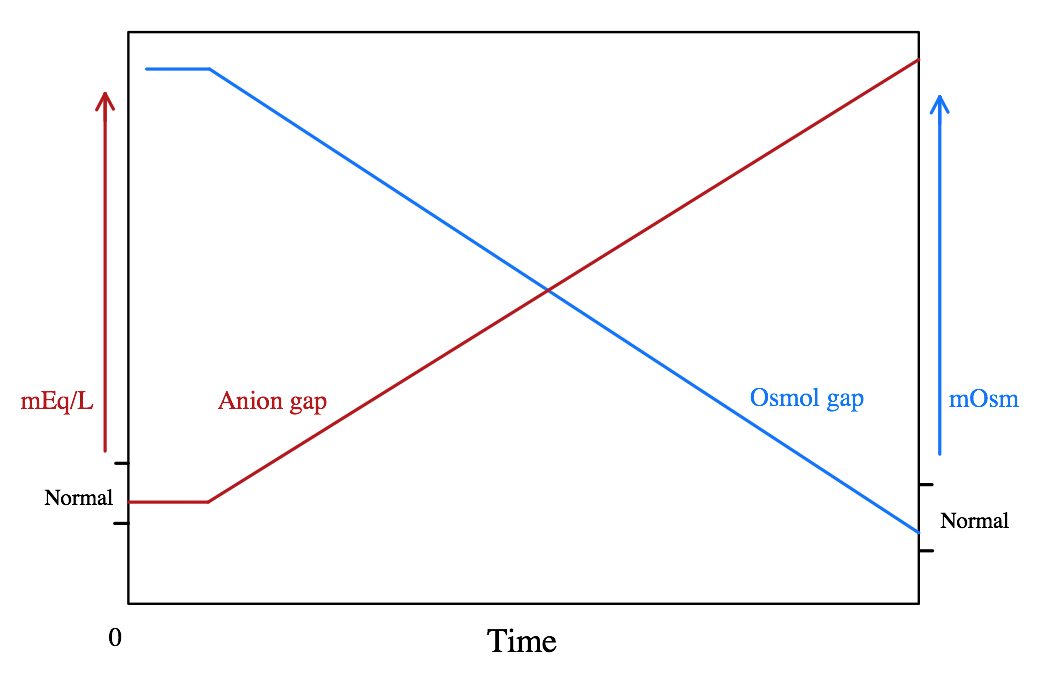

Supplement: Supplementary file 2 [file jetem-5-1-v29-supp2.jpg]
